# Hierarchical Activated Carbon–MnO_2_ Composite for Wide Potential Window Asymmetric Supercapacitor Devices in Organic Electrolyte

**DOI:** 10.3390/mi13111989

**Published:** 2022-11-16

**Authors:** Markus Diantoro, Istiqomah Istiqomah, Yusril Al Fath, Nandang Mufti, Nasikhudin Nasikhudin, Worawat Meevasana, Yatimah Binti Alias

**Affiliations:** 1 Department of Physics, Faculty of Mathematics and Natural Science, Universitas Negeri Malang, Malang 65145, Indonesia; 2 Center of Advanced Materials for Renewable Energy, Universitas Negeri Malang, Malang 65145, Indonesia; 3 School of Physics, Institute of Science, Suranaree University of Technology, Nakhon Ratchasima 30000, Thailand; 4 Department of Chemistry, Faculty of Science, University of Malaya, Kuala Lumpur 50603, Malaysia

**Keywords:** activated carbon, manganese dioxide (MnO_2_), composite, asymmetric supercapacitor

## Abstract

The consumption of electrical energy grows alongside the development of global industry. Generating energy storage has become the primary focus of current research, examining supercapacitors with high power density. The primary raw material used in supercapacitor electrodes is activated carbon (AC). To improve the performance of activated carbon, we used manganese dioxide (MnO_2_), which has a theoretical capacitance of up to 1370 Fg^−1^. The composite-based activated carbon with a different mass of 0–20% MnO_2_ was successfully introduced as the positive electrode. The asymmetric cell supercapacitors based on activated carbon as the anode delivered an excellent gravimetric capacitance, energy density, and power density of 84.28 Fg^−1^, 14.88 Wh.kg^−1^, and 96.68 W.kg^−1^, respectively, at 1 M Et_4_NBF_4_, maintaining 88.88% after 1000 test cycles.

## 1. Introduction

As the performance of electrical energy storage affects the growth of electronic devices, it has recently drawn the attention of researchers. Future energy storage is anticipated to be inexpensive, long-lasting, highly efficient, and environmentally friendly [1]. The lithium-ion battery is one of the energy storage methods that has gained great interest due to high energy density and thermal stability [2]. However, lithium is a toxic material that could highly affect the environment. As an alternative, the supercapacitor has become one of the most promising solutions, with high power density, specific capacitance, rate capability, fast charging, and low environmental pollution [3,4,5]. Commercial supercapacitors are applicable to electronic devices, digital products, and electric cars, and are thus necessary for reliable energy storage.

The supercapacitor is mainly composed of electrodes, electrolytes, and a separator. Supercapacitors commonly use AC and conductive polymers as the active electrode material [6,7]. AC has an affordable price, controllable specific area and a broad pore size distribution [8]. Numerous studies on activated carbon as an electrode material for supercapacitors have shown it can be fulfilled to achieve a specific capacitance of 400 Fg^−1^, 348.2 Fg^−1^, and 286.92 Fg^−1^ in the three-electrode system [9,10,11]. However, only 86.7 Fg^−1^ of specific capacitance can be achieved in the case of coin cell devices [12]. The carbon-based electrode has limited capacitance, which is 200 Fg^−1^ when it turns into a coin cell device, preventing its application range as an energy storage source [13]. As a result, alternative materials are required, such as electrode composites consisting of carbon and transition metal oxides with high pseudocapacitive characteristics [14,15].

A number of metal oxides, including ruthenium oxide (RuO_2_), manganese (III) oxide (Mn_2_O_3_), nickel oxide (NiO), cobalt oxide (CO_3_O_4_), and vanadium oxide (V_2_O_5_), are commonly utilized as supercapacitor electrode materials due to their extremely high theoretical capacitance and charge transfer mechanism [16,17]. However, metal oxides such as RuO_2_ are considered toxic, and their capacitance is still 200 Fg^−1^ [18]. MnO_2_ has a strong possibility of being used as a supercapacitor electrode material due to its significant benefits over other metal oxides, including reversible redox reaction occurring on the surface of the active electrode, high specific capacitance (300–800 Fg^−1^), safety, environmental friendliness, low price, and abundance in nature [19,20,21]. MnO_2_ also has a mesoporous cavity, which is helpful in the process of charge storage and ion transport [22]. Combining MnO_2_ with activated carbon might raise supercapacitors with high capacitance and power density. MnO_2_ nanostructures are one of the constant components that would facilitate synergism to achieve suitable dimensions, crystal structure, crystallinity, and electrolyte selectivity to induce superior capacitance, charge transfer kinetics mechanism, as well as mechanical properties stability and environmental reliability [23]. MnO_2_ has various crystallographic forms, including α, β, γ, δ, and λ, with their own tunnel structures [24,25]. The capacitance properties are heavily influenced by the level of proton or cation intercalation/deintercalation into the MnO_2_ matrix. Some crystallographic forms, such as one-dimensional tunnel structures, appropriate facility gaps for the ions, are anticipated to have particularly high capacitance [26].

In order to optimize MnO_2_’s characteristics, experiments must be conducted to identify the ideal composition of MnO_2_ addition as a composite with AC. Singu et al. experimented with different KMnO_4_ in low molarities around 12 to 50 mM, and the CNT-MnO_2–_25 mM electrode achieved the highest capacitance [27]. Unfortunately, the Singu method cannot be used directly as an electrode material for commercial devices. The synthesis takes a long time, requires expensive materials, and yields a relatively small material. We proposed an effective way to fabricate supercapacitor devices with a commercial AC and MnO_2_, which could be helpful for practical application in an energy storage. We assembled the fabrication of two types of supercapacitors in coin cells (LIR2032) and cylindrical cells (18650) for symmetric and asymmetric assembly. The MnO_2_ was prepared as 10, 15, and 20% in mass. We found that constructing asymmetric supercapacitors increases the maximum operation voltage, specific capacitance, and energy density.

## 2. Materials and Methods

### 2.1. Materials

The chemicals used in this work (from multiple companies) were analytical grade and used without purification: activated carbon (AC, CGC, Bangkok, Thiland), carbon black (CB, Imerys, La Hulpe, Belgium), manganese doxide (MnO_2_, Sigma Aldrich, Burlington, MA, USA), polyvinylidene fluoride (PVDF, Sigma Aldrich, Burlington, MA, USA), dimethylacetamide (DMAc, Sigma Aldrich, Burlington, MA, USA), tetraethylammonium tetrafluoroborate (Et_4_NBF_4_, Gelon, Shandong, China), acetonitrile (ACN, Merck, Darmstadt, Germany). The set of coin cells and cylindrical cells was purchased from TOB machine, Fujian, China.

### 2.2. Synthesis of AC-MnO_2_ Composite

The composite paste was prepared using the ball mill mixing method with a mass ratio of AC-MnO_2_:CB:PVDF is 8:1:1. Briefly, PVDF was dispersed in dimethylacetamide DMAc, which was stirred for 1.5 h, yielding a binder solution. Then, the activated carbon, manganese dioxide (0, 10, 15, 20%), and carbon black were added into the binder solution, forming paste electrode. The paste electrode solution was mixed in a ball mill equipment with 100 g ceramic balls for 48 h. The electrode paste was deposited on an aluminum foil substrate on one side and two sides by using a doctor blade and subsequently dried in an oven at 50 °C for 24 h.

### 2.3. Assembly of the Supercapacitor Devices

One side of the electrode was cut into a circle with a diameter of 2 cm and arranged in a coin cell LIR2032 set with an organic electrolyte 1.0 M Et_4_NBF_4_/ACN and cloth fiber separator. All the electrodes were arranged symmetrically and asymmetrically, with AC-MnO_2_ as the cathode and AC as the anode. The two sides electrode of AC-MnO_2_ were cut into 23 × 4 cm^2^ for the cathode, and AC was cut into 25 × 4 cm^2^ for the anode. Two layers of the separator were cut into 30 × 5.5 cm^2^ and placed between the two electrodes, and then rolled with cylindrical cell equipment. About 10 mL 1.0 M Et_4_NBF_4_ was injected into the cylindrical cell and vacuumed for a while to remove air.

### 2.4. Characterization

The structural information of each material was characterized by an X-ray diffractometer (XRD, PAN Analytical X’Pert PRO, Malvern Panalytical, Worcestershire, United Kingdom) and a Raman spectrometer. The AC, MnO_2_, and film electrode morphology were characterized by scanning electron microscopy (SEM, Inspect S50, FEI Company, Hillsboro, OR, USA) and transmission electron microscopy (TEM, JEM-1400, JEOL, Tokyo, Japan). Porous structures of each material and film composites were characterized by nitrogen adsorption and desorption at 77 K with ASAP-2460 (Micromitics, Hexton, United Kingdom) and Quantachrome 3.0. The specific surface area was calculated using the multi-point Brunauer–Emmett–Teller (BET) method and pore size distribution using Barret–Joyner–Halenda (BJH). For the electrochemical measurements, cyclic voltammetry and electrochemical impedance spectroscopy (CV and EIS) were performed in a two electrode system (PGSTAT302N, Metrohm, Herisau, Switzerland). CV measurements were performed within a voltage window between 0 and 2 V, with the scan rates varied from 10 to 100 mVs^−1^. EIS analysis was performed over a frequency range from 100 kHz to 10 MHz. In addition, electrochemical performance was characterized by galvanostatic charge–discharge (GCD, BTS4000, Neware, Shenzhen, China). GCD cycles were conducted using current densities between 0.1–1.5 Ag^−1^ and with the cut-off voltages of 0–2.6 V.

### 2.5. Calculation

The surface area observed by BET measurement was then calculated using Equation (1) from the t-plot method [28]:

(1) $$\frac{x}{W\left[1-x\right]}=\frac{1}{C\times W_{\mathrm{ml}}}+\left[\frac{C-1}{C\times W_{\mathrm{ml}}}\right]x$$

where W is the mass adsorbed at relative vapor pressure; x = P/Po (P and Po are the actual and saturated vapor pressures of adsorbate, W_ml_ is the needed mass of adsorbate adsorbed on a particular sample to produce a complete monolayer. C represents a constant that expresses the differences in the heat of adsorption of the first and second or higher layers and is the temperature and first layer heat of the adsorption dependent.

The gravimetric capacitance (C, F.g^−1^) was calculated using Equation (2) from the galvanostatic charge–discharge, according to the following Equation (2) [29,30]:

(2) $$C=\frac{4I\Delta t}{m\Delta V}$$

where I is the constant discharge current (A), ∆t is the discharge time (s), m is the mass of active material (g) on two electrodes, and ∆V is the voltage difference (V), excluding the ohmic (IR) drop.

The gravimetric specific energy density (E, Wh.kg^−1^) and power density (P, W.kg^−1^) of the cells were calculated using Equations (3) and (4), respectively [29,30]:

(3) $$E=\frac{1}{8}\frac{C\Delta V^{2}}{3.6}$$

(4) $$P=\frac{E\times 3600}{\Delta t}$$

where C represents the gravimetric specific capacitance of the cell, ∆V corresponds to the voltage change during the discharge process after the IR drop, and ∆t is the discharge time (s).

## 3. Results

**Figure 1:**
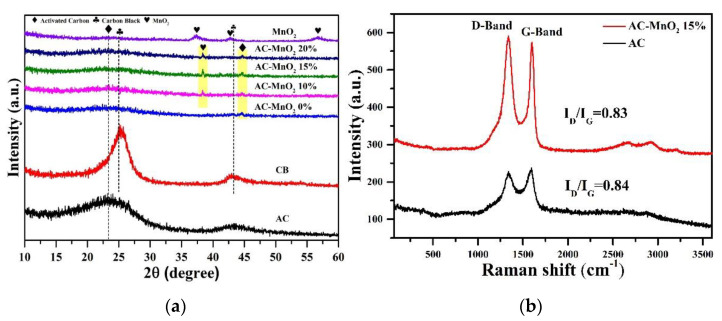
(**a**) X-ray diffraction patterns, and (**b**) Raman spectra of the electrode film with the raw material.

The samples were characterized by X-ray diffraction (XRD), as shown in Figure 1a. According to the X-ray diffraction pattern, MnO_2_ powder has ramsdellite peaks (γ-MnO_2_) at 2θ = 37.381°, 43.827°, and 57.171°, associated with (011), (410) and (420) planes, respectively. However, the AC/MnO_2_ composite electrode films only had one γ–MnO_2_ peak with a hkl (111) plane at 2θ = 38.715°. No obvious peaks were observed, indicating amorphous and poor crystallinity of MnO_2_. However, MnO_2_’s imperfections or poor crystallinity may be advantageous when employed in supercapacitors as the path way for ion migration between the materials [31].The two-dimensional layer structure of MnO_2_ makes it suitable for the intercalation and deintercalation of ionic electrolytes, improving its capacitive characteristics [32]. The orthogonal unit cell with a space group of 62 (Pnma) was indicated by the diffraction peaks of γ-MnO_2_, which were consistent with the standard data file COD 96-210-5791. Lattice parameters for MnO_2_ nanoparticles are a = 9.323 Ǻ, b = 4.453 Ǻ, and c = 2.848 Ǻ, with α = β = γ = 90°, as shown in Figure 1a.

The AC-MnO_2_ 0% electrode composites produced weak peaks at 2θ = 23.69° (002), 25° (002), and 44.28° (110), where the peak intensity was lower and tended to broaden, showing that carbon has an amorphous structure [13,33]. Activated carbon has an amorphous structure, which contributes to its high specific surface area and boosts its specific capacitance [34]. However, MnO_2_ has a reasonably specific capacitance range, measuring between 140.30 and 240 Fg^−1^ [35]. Therefore, it is expected that increased capacitance can be obtained by synthesizing the AC/MnO_2_ composite by modifying the weight mass of MnO_2_. Different AC/MnO_2_ composites showed the diffraction peak’s intensity at 2θ = 38.715°. Despite the low percentage of MnO_2_ mass addition, the intensity of these peaks did not seem to increase significantly. In addition, the XRD diffraction pattern was dominated by carbon peaks throughout 80% of the electrode samples made of activated carbon and carbon black. The percentage of MnO_2_ and activated carbon needs to be further characterized, specifically by utilizing SEM-EDX in the discussion of the following paragraphs.

We employed Raman spectroscopy to investigate the crystallinity of graphitic planes and disordered regions of the activated carbons and their composite with MnO_2_–15%. The Raman spectra of all as-prepared activated carbons and the AC–MnO_2_ 15% composite in Figure 1b show two distinct peak positions at 1341.06 cm^−1^ and 1593.74 cm^−1^, which correspond to the D (vibration of disordered sp^3^ carbon) and G bands (ordered sp^2^ carbon), respectively [36]. The integral ratio (I_D_/I_G_) is used to evaluate the degree of the structural disorder compared to a perfect graphitic structure [37]. In this study, ratios of AC and AC–MnO_2_ 15% reached 0.84 and 0.83. Coincidentally, another research has a similar integral ratio results. Previous work reported the integral ratio of activated carbon was 1.11. When the activated carbon turned into activated amorphous carbon (AAC), the integral ratio decreased up to 0.84. Based on the I_D_/I_G_ ratio, AAC has a higher graphitization degree, creating an opportunity for improved their electrical conductivity [38,39]. However, the I_D_/I_G_ ratio of 15% AC–MnO_2_ was lower than AAC, leading to better performance

**Figure 2:**
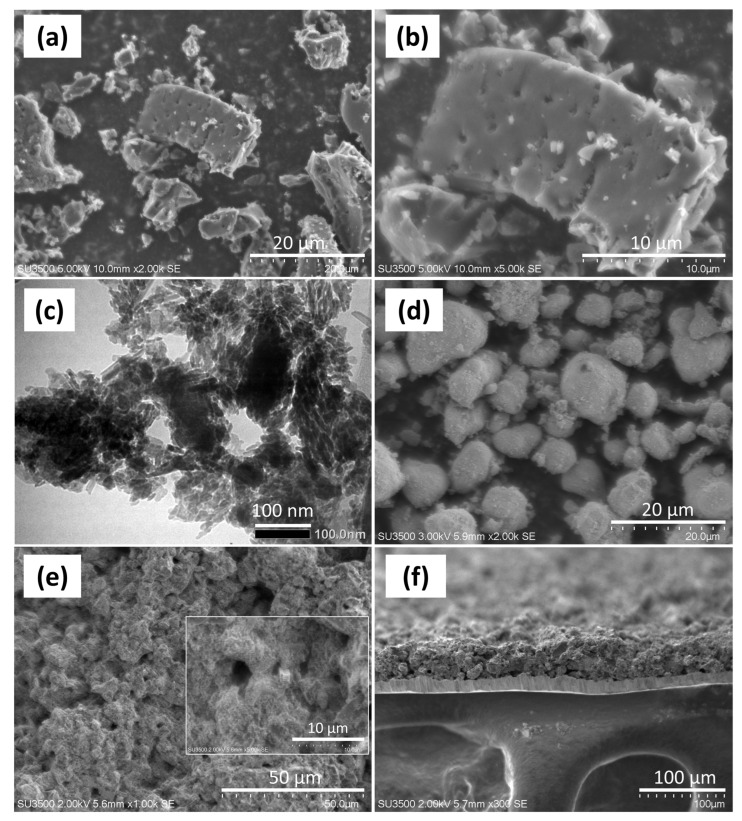
Morphology based on (**a**,**b**) SEM images of activated carbon powder, (**c**) TEM images of MnO_2_ (inset: 150,000×), SEM images of (**d**) MnO_2_, (**e**) AC–MnO_2_ 15% electrode film (inset: 5000×), (**f**) cross section electrode film.

The primary component used in creating the electrodes for supercapacitors was activated carbon. As a result, activated carbon made up roughly 80% of the supercapacitors’ primary materials. SEM analysis with various magnifications, as shown in Figure 2a,b, was used to validate the presence of pores in the activated carbon. Figure 2b shows apparent clear pores of activated carbon on the surface at a magnification of 5000 times. It makes activated carbon with a wide surface area and a pore structure that allows ions to move more efficiently during the energy storage process [40]. Based on TEM characterization of MnO_2_ nanoparticles at different magnifications of 40,000 times and 150,000 times (inset), Figure 2c depicts the shape of the particles. The morphology of γ–MnO_2_ appears to be slightly needle-shaped, and some particles are spherical at a magnification of 150,000 times. The γ–MnO_2_ nanoparticles aggregated at a magnification of 40,000, which is shown as a solid black region. Nanoparticles agglomeration could decrease the electroactive surface area of the nanocomposite, which can actually reduce the electrochemical performance [41]. The surface area of MnO_2_ is quantitatively proven in the BET measurement in the next section. MnO_2_ is non-uniformly sized and has spherical morphology. The γ-MnO_2_ needle measures 7.24 nm in length. The MnO_2_ nanoparticles have a particle size of 4.2 nm. In order to determine the shape of γ–MnO_2_ based on its surface, SEM analysis is required. In contrast to the TEM data, the SEM results in Figure 2d depict the shape of the γ-MnO_2_ nanoparticles as spherical.

Based on the SEM analysis in Figure 2e, it is possible to estimate the shape of the AC–MnO_2_ 15% supercapacitor electrodes in different magnifications. According to Figure 2e, activated carbon pieces predominate on the electrodes. At 5000× magnification (inset Figure 2e), activated carbon possesses holes that are particularly conducive to the transport of electrolyte ions into the electrode, improving the electrode’s electrochemical performance [29,42]. As a result of the interaction between activated carbon and PVDF (binder), the electrode surface appeared to have agglomerated in numerous places [43]. The thickness of the electrode is also crucial in determining the electrochemical performance of the supercapacitor device because it can boost current and energy density [44,45]. However, overly thick electrodes might raise the resistance and reduce the voltage delivered to the electrodes, reducing capacitance [46]. Therefore, a suitable thickness must be determined based on the electrode constituent material [47]. The thickness of the electrode can be seen in the cross section at 300 times magnification in Figure 2f. The thicknesses of the AC–MnO_2_ 15% electrodes is 43.50 μm. Both have similar thicknesses but distinct variances in the curvature of the electrode surface, with AC–MnO_2_ 15% being flatter than AC–MnO_2_ 10%. Due to their lower percentage than activated carbon, MnO_2_ nanoparticles were also less noticeable, according to the SEM data.

**Figure 3:**
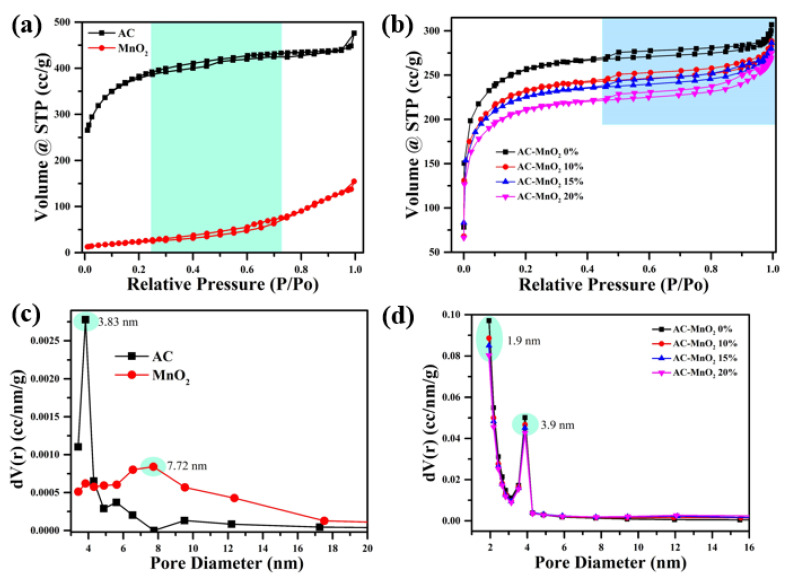
(**a**) N_2_ adsorption/desorption isotherms at 77 K of raw material and (**b**) composite, (**c**) pore size distributions of raw material, and (**d**) composite.

A crucial factor in developing high-performing supercapacitors is the pore size of the activated carbon. Porosity is essential in determining electrochemical performance, which practically impacts ion electrolytes’ interaction with various transport paths in the electrode surface [48,49]. As a result, the porosity of the electrode should be determined. The porous structure of the raw materials and composites was investigated using nitrogen adsorption–desorption measurements at 77 K. Figure 3a,b shows the adsorption–desorption isotherm of AC, MnO_2,_ and the composite film electrode AC-MnO_2_ 0–20%. Activated carbon and MnO_2_ raw material isotherm curves in Figure 2a could be classified as type I and IV, typical of microporous materials [29]. The strong adsorption at 0.1 relative pressure, followed by a relatively open knee before reaching equilibrium, suggested microporous carbon with a wide range of micropore diameters [50]. Figure 2b shows the N_2_ adsorption–desorption isotherms of the composite.

According to the International Union of Pure and Applied Chemistry (IUPAC) classification, all three forms of the composite display type IV isotherms. At medium relative pressure (P/Po = 0.5–0.8), an apparent hysteresis loop shows the presence of abundant mesopores [51]. Furthermore, no adsorption increase in the isotherm was seen at high relative pressure (P/Po = 0.9–1.0), demonstrating the unavailability of macropores [29]. The essentially mesoporous structure of both raw materials and composites is confirmed by its pore size distribution, which shows that the majority of its pores are in the mesopores range (>2 nm) (Figure 3c,d) [52]. Furthermore, the pore size distribution curves show that both are primarily formed of mesoporous structure, with the pore size concentrated in 3.83–7.72 nm, indicating that the sample is primarily composed of mesopores. This mesoporous cavity is helpful in the process of charge storage and ion transport. Table 1 shows the specific microstructure data of the samples where the surface area was calculated using Equation (1). The results demonstrate that activated carbon exhibits a more significant specific area (1227.96 m^2^g^−1^), and a greater pore volume (0.73 cm^3^g^−1^) than the MnO_2_, resulting in better interaction with the electrolyte and faster ion movement. However, after combining AC and MnO_2_, the surface area and pore volume dropped, although the loss was not significant. This clearly shows that MnO_2_ aggregation, as we mentioned previously in the TEM images, influences the surface area of the composite. Although MnO_2_ doping reduces the particular surface area to several amounts, the pseudocapacitance produced by MnO_2_ could even boost the specific capacitance of porous carbon-based materials [50].

The large surface area and pore structure of porous carbon facilitate the ability of ions to contribute to the energy storage process. Ion transfer will happen more quickly because of these pores’ large adsorption area for electrolyte ions [53]. To allow ions to enter and be absorbed into the activated carbon pores, which will increase the amount of electrical energy stored, the diameter of the pores should match that of the electrolyte ions. The distribution curve revealed that activated carbon’s pore size was 3.83–3.9 nm. The diameter of the Et_4_N^+^ ion is 0.343 nm, while that of the BF_4_– ion is 0.229 nm [54]. This smaller ion diameter size may be absorbed fast into the pores of the activated carbon, resulting in superior supercapacitor performance [55]. Lee et al. conducted a similar investigation on the change in pore size with electrolyte ions in 2021. Lee et al. employed activated carbon from coconut shells and LiNO_3_ as electrolytes. The pore size of the activated carbon produced is more significant than 0.40 nm. The Li^+^ ion, on the other hand, has an ion diameter of 0.38 nm, while the NO_3_^−^ ion has an ion diameter of 0.34 nm. An asymmetric coin cell supercapacitor device with a specific capacitance of 88 Fg^−1^, an energy density of 48.9 Wh/kg, and a power density of 1 kW/kg, at a current density of 1 Ag^−1^, can be created by combining pores and electrolytes [56].

Supercapacitor performance is determined not only by the electrode material but also by the electrolyte and separator. As a crucial component, the electrolyte primarily influences the voltage window and rate capability of the energy storage device, therefore improving its energy density and power density [57]. Liquid electrolytes have been considered as a promising electrolyte for supercapacitors, owing to their properties, such as being non-flammable [33]. Organic electrolytes such as Et_4_NBF_4_ are frequently employed because they may be used in low-cost current collectors such as aluminum, with a voltage range of 2.5–2.8 V [58]. In addition, the separator is essential in defining the supercapacitor’s performance capability. The separator must be thin, porous, and have an excellent dielectric substance [59]. One of the widely used separators is cloth fiber because it is thin and allows ions to diffuse quickly.

**Figure 4:**
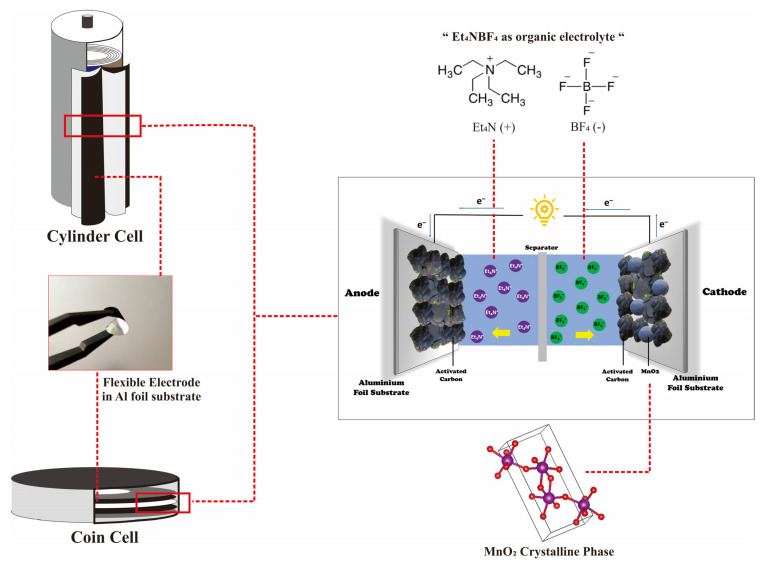
The schematic mechanism illustration of fabricated asymmetric supercapacitor devices based on activated carbon as negtive electrode and AC–MnO_2_ 15% as negative electrode in an Et_4_NBF_4_ organic electrolyte.

**Figure 5:**
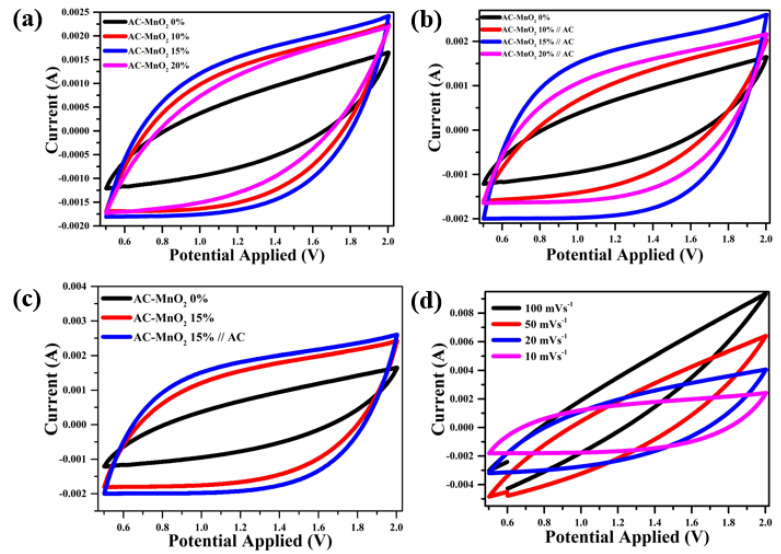
Cyclic voltammograms of (**a**) symmetric supercapacitors of AC-MnO_2_ 0–20%, (**b**) asymmetrical supercapacitors of AC-MnO_2_ 0–20%//AC, (**c**) comparison between the symmetric and asymmetrical of AC–MnO_2_ 15% and (**d**) AC–MnO_2_ 15%//AC at a scan rate of 10–100 mVs^−1^.

The performance of electrodes was considered by fabricating coin cells with two electrodes, symmetric and asymmetrical. The voltage range used in this preliminary investigation was 0.5–2 V, with scan rates of 100, 50, 20, and 10 mVs^−1^. The three curves are all the same shape, semi-rectangular (quasi-rectangular), indicating that the electrode is of the EDLC type, where the mechanism is described in Figure 4 [60,61]. This is due to the electrode’s composition, which is ±80% dominated by carbon material, with the carbon material incorporated in the EDLC type [62]. The CV curve in Figure 5a–c is generated at a scan rate of 10 mVs^−1^ and was used to compare electrode performance for each weight mass. The symmetrical CV curve in Figure 5a covers the entire sample, which is 0% to 20% MnO_2_. The curve region occupied by the AC-MnO_2_ 0% electrode is less than that of the electrode containing MnO_2_. Although both curves have nearly uniform shapes, the AC- MnO_2_ 15% electrode has the largest curve area. Figure 5b shows the same result for the asymmetric cell. Figure 5a,b distinguish that the asymmetric cell curve AC-MnO_2_ 15%//AC has a more significant area than the symmetric one. Figure 5c shows the slight difference between the symmetric and asymmetrical curves. However, when compared to the electrode curve obtained without the addition of MnO_2_, the increase in the area of the curve obtained is rather significant. The rise in the area of this curve implies that the electrode can store more charge, increasing the specific capacitance [63]. These results suggest that, while the CV curve depicts EDLC characteristics, MnO_2_ also improves the contribution of EDLC performance [64]. Figure 5d depicts the AC-MnO_2_ 15% asymmetrical CV curve at various scan rates. The curve is exceptionally well constructed without being interrupted at a scan rate of 50–100 mVs^−1^, suggesting an increase in electron conduction in the electrode, which supports fast charge diffusion, and thus the capacitive performance of the electrode is excellent and stable [32,65]. However, when viewed from the entire curve, there is a shift in shape as the scan rate increases. It remains semi-quasi-rectangular at scan rates of 10 and 20 mVs^−1^. When the scan rate is 50–100 mVs^−1^, the curve becomes more pointed at the tip due to insufficient diffusion of electrolyte ions [66,67]. As a result, the electrochemical reaction fails because electrolyte ions cannot access the pores of the active material at high scan rates [68].

**Figure 6:**
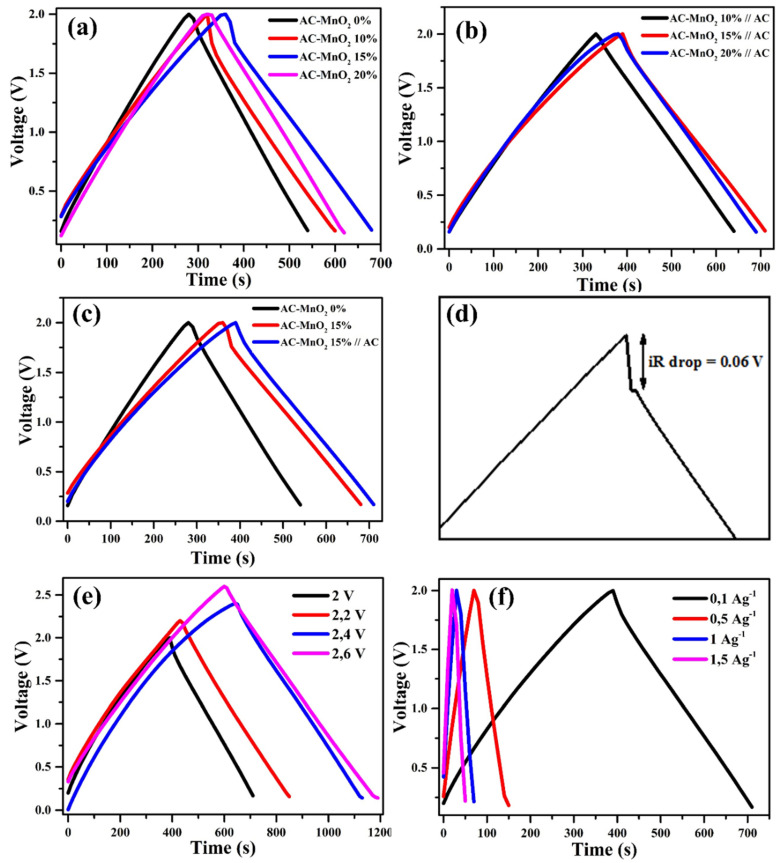
Electrochemical performance based on GCD curve of (**a**) symmetric, (**b**) asymmetric coin cell supercapacitor, (**c**) comparison for both, (**d**) iR drop of an asymmetric supercapacitor, (**e**) charge–discharge behavior of asymmetric supercapacitor on 22.6 V, (**f**) and different current densities at 0.1–1.5 Ag^−1^.

The charge–discharge curve in Figure 6a–c has a triangular shape that slightly changes at the peak due to the reversible redox reaction occurring on the surface of the active electrode, indicating that the AC-MnO_2_ composite has good electrochemical reversibility with capacitance derived from the combination of EDLC and pseudocapacitive capacitance [69]. This imperfect curve shape is also caused by the voltage drop (IR drop). A sudden voltage drop, commonly known as an IR drop, as shown in Figure 6d, occurs when a supercapacitor changes from a charged to a discharged state as a result of a combination of electrode ohm resistance, electrolyte, ion transfer in the electrode material and the contact voltage in the electrochemistry system [70]. The electrode owns the most significant IR drop without MnO_2_, which is 0.1 V. This indicates that the AC-MnO_2_ 0% electrode has a more excellent internal resistance than the other electrodes. The AC–MnO_2_ 15%//AC electrode has the smallest IR drop of 0.06 V, indicating good electrochemical performance and electrical conductivity because ion transfer occurs quickly [71].

The gravimetric capacitance of the supercapacitor electrode was calculated using Equation (2). Figure 6a shows the charge–discharge curve of a symmetric coin cell. An increase in MnO_2_ gives rise to an increased charge time following a slight reduction exhibiting by 20%. As a result, the longest discharge time is recorded by AC–MnO_2,_ 15%, almost 700 s. The charge–discharge of asymmetric assembly is shown in Figure 6b. The same result is also shown by the AC–MnO_2_ 15%, with the longest discharge time exceeding the symmetric coin cell, which is more than 700 s. The difference in the curve between the coin cell baseline (AC–MnO_2_ 0%), symmetric and asymmetric, can be seen in Figure 6c. The curve shows an increase in discharge time when MnO_2_ exists and the asymmetric configuration. The capacitance, energy density, and power density of each cell are shown in Table 2.

The specific capacitance in Table 2 shows that the most optimum supercapacitor performance is owned by the electrode AC–MnO_2_ 15% with a gravimetric capacitance of 79.43 Fg^−1^. This is due to a reduction in internal resistance and a faster transfer of electrolyte ions when MnO_2_ is added 15% [72]. A slight decrease to 74.32 Fg^−1^ by 20% MnO_2_ is believed to be the resistance increase in the electrode inhibiting the reaction between MnO_2_ and the electrolyte [73]. The addition of MnO_2_ also impacts the electrical conductivity of the electrode material. With more MnO_2_ added, the electrode electrical conductivity will decrease and reduce the supercapacitor’s performance [68]. The data in Table 2 also show an increase in performance in asymmetric supercapacitor cells. The cathode in the asymmetric cell is AC-MnO_2_ 15%, where Et_4_N^+^ ions are generated and fed from/to the electrode material.

In contrast, the AC at the anode functions to run the EDLC system on its surface. The asymmetric coin cell’s specific capacitance, energy density, and power density reach their optimum limits at the mass percent MnO_2_ of 15%, in line with the symmetric cell. AC–MnO_2_ 15%//AC can have a specific capacitance of 81.63 Fg^−1^, which is none other than 2.76% higher than AC–MnO_2_ 15% symmetric coin cell. The increase in specific capacitance is much more significant than the baseline, reaching 15.44%.

Asymmetric supercapacitors perform at a higher potential window when compared to symmetric [74]. A voltage variation of 2–2.6 V and a current density of 0.1–1.5 Ag^−1^ was carried out to determine the impact of the asymmetrical electrode. The asymmetric cell can be supplied with a voltage of up to 2.6 V with an excellent charge–discharge curve, as shown in Figure 6e. The larger potential window greatly enhanced the performance of the supercapacitor. The more significant the voltage used, the longer the discharge time will be. According to Equation (2), the gravimetric capacitance of the asymmetric cell will arise with increasing voltage. The gravimetric capacitance of AC–MnO_2_ 15%//AC in operating voltage of 2, 2.2, 2.4, and 2.6 is 81.63, 85.02, 89.43, and 98.48, respectively. The highest capacitance achieved at a voltage of 2.6 V is 98.45 Fg^−1^, with energy density and power density of 21.07 Wh/kg and 103.98 W/kg, respectively. Electrolytes also play an essential role in the electrochemical performance and determining the cost of supercapacitor devices. Several electrolytes currently being developed include liquid ionic, solid, and organic electrolytes. Ionic liquid electrolytes have low ionic conductivity and high viscosity, increasing the equivalent series resistance (ESR), which could slow down the supercapacitor performance rate [58]. Solid electrolytes have low ionic conductivity at room temperature, although the potential difference is quite significant [75,76]. Currently, organic electrolyte-based supercapacitors are leading the commercial market because they offer a more considerable and stable potential difference (≥2.5 V) [77]. Based on the data above, it can be concluded that using a combination of EDLC with pseudocapacitive materials and increasing the potential difference through an asymmetric system, and also organic electrolyte, is one way to increase the specific capacitance, energy density, and supercapacitor power density [78].

In contrast to the voltage, the greater current density will make the discharge time occur very quickly, as shown in the form of a charge–discharge curve in Figure 6f. Different current densities were also carried out on asymmetric cells AC–MnO_2_ 15%//AC to determine the stability of the electrodes. The greater the current density, the faster the discharge time of the supercapacitor. The shape of the curve will be narrower as the current density increases, as shown in Figure 6f. However, there is no change in the shape of the curve, indicating good electrochemical storage [68]. The current density difference also affects the supercapacitor’s gravimetric capacitance. The gravimetric capacitance of AC–MnO_2_ 15%//AC cells at 0.1, 0.5, 1, 1.5 Ag^−1^ are 81.63, 79.83, 76.28, and 73.56 Fg^−1^, respectively. The decrease in capacitance as the current density increases is due to electrolyte ions that do not have enough time to diffuse across the sample surface at high current densities [79].

The stability of the electrochemical performance of asymmetric cell AC–MnO_2_ 15%//AC can be determined through the charge/discharge characterization carried out up to 1000 cycles at a current density of 1 Ag^−1^, as shown. Asymmetric coin cell AC–MnO_2_ 15%//AC could hold up to 91.97% after 1000 test cycles. Good cycle stability indicates that the Faradaic electrode and electrolyte reaction is highly reversible [80]. Based on the research results using the coin cell, above, the AC–MnO_2_ 15%//AC electrode is packaged as a cylindrical cell to obtain higher discharge energy. It aims to produce supercapacitors that are ready to be commercialized. A comparison of the results of this study with previous studies has been summarized in Table 3.

Table 3 compares research on different materials, ranging from nickel-based composites, rGO, MnO_2_, and activated carbon, to graphene packaged in the form of coin cells. Our work shows a better performance, where the capacitance can reach 98.45 Fg^−1^ at a voltage of 2.6 V, an energy density of 21.07 Wh/kg, and a power density of 103.98 W/kg. Comparison of energy density and power density was also carried out with potentiostatic (PS)+ potentiodynamic (PD) (MnO_2_/Ni)//AC (7.7 Wh/kg at 600 W/kg) studies [87], a-MnO_2_@d-MnO_2_//AC (12.9 Wh/kg at 230 W/kg) [81], NiCo_2_O_4_–MnO_2_//activated graphene (9.4 Wh/kg at 175 W/kg) [88], MnO_2_-modified diatomite (3.75 Wh/kg at 250 W/kg) [89], AC/juglone//AC (12 Wh/kg at 180 W/kg) [90], MnO_2_-carbon black//AC (11 Wh/kg at 50 W/kg) [91]. From all these studies, the value of energy density and power density in this study is still higher than the results of previous studies. So, it can be concluded that this research has been successful.

**Figure 7:**
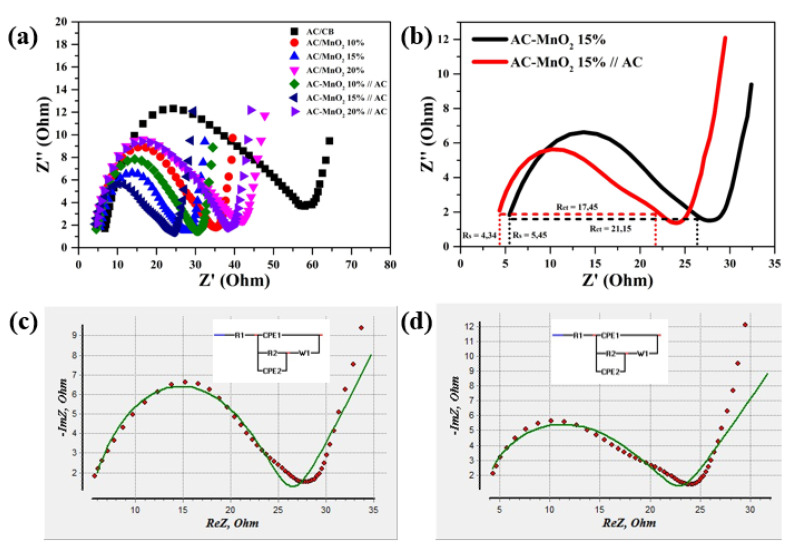
Nyquist plot of (**a**) symmetric and asymmetric supercapacitors, (**b**) comparison of both of them in the 15% weight mass ratio of MnO_2_, (**c**) fitting plot of symmetric, and (**d**) asymmetric supercapacitors.

Electrochemical impedance spectroscopy (EIS) is one of the fundamental techniques to find information related to ion diffusion and electron transfer at the electrode surface [92]. The shape of the Nyquist plot in Figure 7a approximates the shape of a semicircle with different diameters in each sample. This semicircle shape shows charge transfer and double-layer phenomena in the area between the electrolyte and the electrode [93]. The diameter in the semicircle area is called the charge transfer resistance (Rct), which is the electron transfer resistance between the electrode and electrolyte interface [94]. The two most minor diameters are owned by the asymmetric cell AC–MnO_2_ 15%//AC and the symmetric cell AC–MnO_2_ 15%, respectively 17.45 and 21.15. The smaller the diameter, the faster the charge transfer kinetic ability [95]. A large Rct (R2) indicates an increase in resistance due to ion mobility in the electrode pores. The Rct is very different from the baseline Rct, seen from the vast baseline semicircle. When the electrode is added with MnO_2_ material, the semicircle begins to shrink. The decrease in semicircle diameter is due to the high MnO_2_ electrical conductivity. As a result, the semicircle area gets bigger (Rct). The intercept on the *X*-axis is known as the equivalent series resistance (ESR) and is denoted by Rs (R1). Rs (R1) denotes the effect of electrolytic resistance between electrode and separator [84,85]. As seen in Figure 7b, the ESR or Rs of the asymmetric cell AC–MnO_2_ 15%//AC (4.34) is smaller than the asymmetric cell AC–MnO_2_ 15% (5.45). This smaller value of Rs indicates a lower electrical resistance. In short, the smaller Rs and Rct, the better the supercapacitor’s performance. The comparison of the Rs and Rct of all samples is shown in Table 4.

However, Rs and Rct from the fittings show that the asymmetric cell has a lower resistance than the symmetric cell. The data in Table 4 present the symmetric Rs and Rct coin cells of 4.9748 and 22.014, respectively, with an error of 4.2149% and 2.2726%. In contrast, the asymmetric cell has an error of Rs and Rct of 1.006% and 2.2773%, respectively. The fitting results, as shown in Figure 7c,d also give the CPE and the CPE coefficient (α/β) whose values are less than equal to 1 and more than 0 [96]. When the value is below 1 then the electrode is resistive. On the other hand, when the coefficient of CPE is exactly 1, the electrode is classified as capacitive [97]. Therefore, the coefficient is interpreted in terms of physical properties, such as the ability to generate specific capacitance. The CPE coefficient in Table 4 is denoted by the symbol α, where the asymmetric cell CPE coefficient is 1, which is greater than the symmetric cell of 0.32. Thus, it can be concluded that, based on the EIS results, asymmetric cells have better performance than symmetric ones.

**Figure 8:**
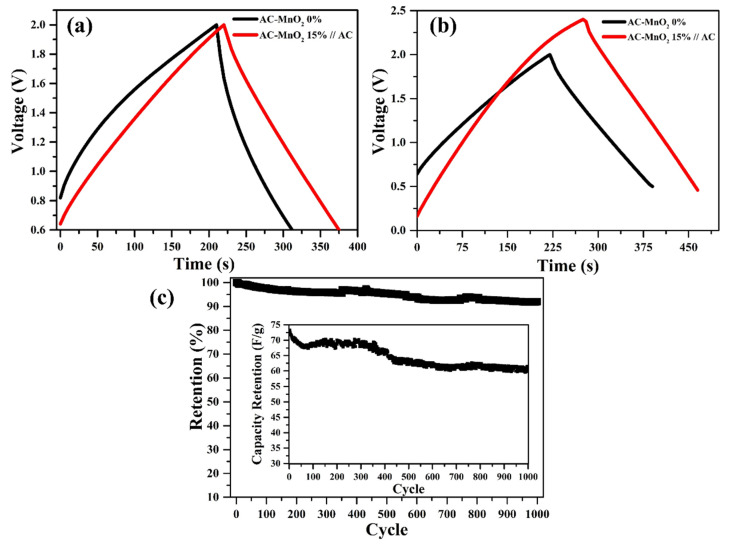
Charge–discharge curves of (**a**) cylindrical cell symmetric AC-MnO_2_ 0% and asymmetric AC–MnO_2_ 15%//AC electrode, (**b**) each cell is measured on different voltage, (**c**) cycle life retention tested at a current density of 1 Ag^−1^ for 1000 times.

In this research, the electrodes were also packed in a cylindrical cell, and then GCD was carried out using a Neware instrument. The GCD of cylindrical cells is shown in Figure 8. Cylindrical cell fabrication was carried out using two types of electrodes, i.e., AC-MnO_2_ 0% and AC–MnO_2_ 15% electrodes arranged asymmetrically at the same voltage of 2 V. Based on the charge–discharge curve in Figure 8a, asymmetric cylindrical cell AC–MnO_2_ 15%//AC has a longer discharge time than without MnO_2_, the same results as in the form of a coin cell. The only difference is that the cylindrical cell discharge time is faster than the coin cell. In addition, when the voltage is increased to 2.4 V, as shown in Figure 8b, the discharge time is longer than when using a 2 V. When compared to a coin cell at a voltage of 2.4 V, cylindrical cells also have a lower discharge time. Decreasing discharge time affects the capacitance, energy density, and supercapacitor power density. The cylindrical cell supercapacitor AC-MnO_2_ 0% has a capacitance of 59 Fg^−1^ with an energy density of 3.49 Wh/kg and a power density of 101.86 W/kg. While the asymmetric cylindrical cell AC—MnO_2_ 15%//AC has a capacitance of 77.7 Fg^−1^ with an energy density of 5.46 Wh/kg and a power density of 115.87 W/kg. When the voltage increases to 2.4 V, the capacitance, energy density, and power density increase, which are 84.28 Fg^−1^, 14.88 Wh/kg, and 96.68 W/Kg, respectively. Coin and cylindrical cells are the difference in discharge time and discharge energy, shown in Table 5.

Energy discharge is the energy accumulation from an energy storage device until it reaches the lowest voltage limit (cut-off voltage). After that, the device will release energy in a certain period so that the unit of energy discharge is mWh. According to Table 5, the energy discharge of a coin cell is very small, different from a cylindrical cell. Based on the above definition of energy discharge, the energy produced by a cylindrical cell is much greater than that of a coin cell. This is undoubtedly since the surface area of the electrodes in a cylindrical cell is much larger than that of a coin cell, so the resulting cylindrical cell can be used for large enough devices. Table 5 can be used to see the difference in the parameters of the GCD test results between coin cells and asymmetric cylindrical cells AC—15% MnO_2_//AC at a voltage of 2.4 V.

Based on the capacitance and energy density in Table 5, there is a decrease when the electrode is packaged as a cylindrical cell. The most significant factor influencing its performance is pressure. The pressure during the rolling process can affect the performance of the supercapacitor because as the pressure increases in the supercapacitor fabrication process, the two electrodes are in perfect contact. As a result, there is an increase in the diffusion of ions entering the pores, which will increase the electrochemical reaction in the cell [98,99]. However, compared to the study by Lee (2018), which obtained 63 Fg^−1^ using Li_4_Ti_15_O_12_ material, this study is still much better, with results of 84.28 Fg^−1^ [100]. As shown in Figure 8c, the best retention capacitance is reached by the asymmetric cell AC–MnO_2_ 15%//AC, which survived up to 88.88% after 1000 test cycles.

## 4. Conclusions

A supercapacitor is environmentally friendly energy storage. The performance of supercapacitors can be improved by combining active materials and oxides transition metals. In this research, composite electrode material based on activated carbon (AC) with a mass variation of MnO_2_ has been successfully synthesized. The electrode owns the most optimum electrochemical performance of the supercapacitor, with the addition of 15% MnO_2_ with a gravimetric capacitance of 79.43 Fg^−1^. The energy and power density values obtained can reach 9.07 Wh/kg and 85.43 W/kg. Asymmetric coin cells have better electrochemical performance than symmetric coin cells. The asymmetric coin cell can have a specific capacitance of 98.45 Fg^−1^ at 2.6 V. The energy and power density obtained is 21.07 Wh/Kg and 103.98 W/Kg, respectively. Asymmetric coin cells have better electrochemical performance than asymmetric cylindrical cells. Asymmetric cylindrical cells have gravimetric capacitance, energy density, and power density of 84.28 Fg^−1^, 14.88 Wh/Kg, and 96.68 Wkg, respectively, which can hold up to 88.88% after 1000 test cycles.

## Figures and Tables

**Table 1:** The BET surface area, total pore volume, and pore structure parameters of raw material and film composites.

Sample	S_BET_ (m^2^g^−1^)	V_total_ (cm^3^g^−1^)	Pore Size (nm)
Activated Carbon (AC)	1227.96	0.73	3.83
MnO_2_	99.91	0.23	7.72
AC–MnO_2_ 0%	808.22	0.46	3.9
AC–MnO_2_ 10%	784.02	0.44	3.9
AC–MnO_2_ 15%	727.13	0.43	3.9
AC–MnO_2_ 20%	666.28	0.42	3.9

**Table 2:** Electrochemical parameter of symmetric and asymmetric supercapacitor.

Sample	Gravimetric Capacitance (Fg^−1^)	Gravimetric Energy Density (Wh.kg^−1^)	Gravimetric Power Density (W.kg^−1^)
AC–MnO_2_ 0%	70.71	8.53	69.59
AC–MnO_2_ 10%	73.91	8.83	74.61
AC–MnO_2_ 15%	79.43	9.07	85.44
AC–MnO_2_ 20%	74.32	8.85	72.01
AC–MnO_2_ 10%//AC	74.50	8.94	78.59
AC–MnO_2_ 15%//AC	81.63	9.21	70.83
AC–MnO_2_ 20%//AC	77.31	9.64	86.75

**Table 3:** Comparative electrochemical performance based on previous research.

Cathode	Anode	Electrolyte	Gravimetric Capacitance (F/g)	Gravimetric Energy Density (Wh/kg)	Gravimetric Power Density (W/kg)	Retention	Ref.
α-MnO_2_@-δ-MnO_2_	AC	1 M Na_2_SO_4_	28.9	12.9	230	73% (10,000)	[81]
MnO_2_/SHAC	SHAC	1 M Na_2_SO_4_	49.2	46.2	3679	80.4% (5000)	[82]
NiCo_2_O_4_	AC	2 M KOH	52.3	21	424.5	99.3% (5000)	[83]
Graphene hydrogel	BDTD-rGO	1 M H_2_SO_4_	54	9.52	450	81.3% (5000)	[84]
rGO/C/MnO_2_	AC	3 M KOH	59.5	21.2	190	72% (2500)	[85]
MnO_2_/Carbon Cloth	AC	1 M Na_2_SO_4_	67.8	18.46	699.54	97.3% (2000)	[86]
AC-MnO_2_ 15%//AC	AC	1 M Et_4_NBF_4_	98.45	21.07	103.98	91.97% (1000)	This work

**Table 4:** Fitting parameter EIS of cell symmetric and asymmetric AC/MnO_2_ 15% supercapacitors.

Sample	CPE	Rs	α	CPE 2	Rct	α	W
AC–MnO_2_ 15%	9.0534 × 10^−6^	4.9748	0.8021	5.1498 × 10^−6^	22.014	0.32438	6.2736
AC–MnO_2_ 15%//AC	27.741 × 10^−6^	3.1401	0.4643	3.954 × 10^−7^	19.976	1	6.9894

**Table 5:** Energy discharge comparison of coin cell and cylindrical cell.

Cell	Gravimetric Capacitance (F/g)	Gravimetric Energy Density (Wh/kg)	Gravimetric Power Density(W/kg)	Energy Discharge (mWh)
Coin Cell	89.43	16.34	100.04	0.244
Cylindrical Cell	84.28	14.88	96.68	18.062

## Data Availability

The data presented in this study are available upon request from the corresponding author.
